# Trends in primary surgery and overall survival in non-metastatic anal cancer: a population-based analysis

**DOI:** 10.1093/oncolo/oyag148

**Published:** 2026-04-17

**Authors:** Chan Shen, Madison Conces, Kristen Ciombor, Jennifer Dorth, Joshua E Meyer, Timothy M Geiger, Aimal Khan, Jeneth Aquino, Natalie A Lockney, A Bapsi Chakravarthy, Jennifer Whisenant, Al B Benson, Cathy Eng

**Affiliations:** College of Medicine, Departments of Surgery and Public Health Sciences, Pennsylvania State University, Hershey, PA 17033, United States; University Hospitals Seidman Cancer Center, Case Western Reserve University, Cleveland, OH 44106, United States; Division Hematology and Oncology, Vanderbilt-Ingram Cancer Center, Nashville, TN 37232, United States; Department of Radiation Oncology, Seidman Cancer Center, University Hospitals Cleveland Medical Center, Cleveland, OH 44106, United States; Department of Radiation Oncology, Fox Chase Cancer Center, Philadelphia, PA 19111, United States; Section of Colon and Rectal Surgery, Division of General Surgery, Vanderbilt University Medical Center, Nashville, TN 37232, United States; Section of Colon and Rectal Surgery, Division of General Surgery, Vanderbilt University Medical Center, Nashville, TN 37232, United States; Vanderbilt University School of Medicine, Nashville, TN 37232, United States; Department of Radiation Oncology, Vanderbilt University Medical Center, Nashville, TN 37232, United States; Department of Radiation Oncology, Vanderbilt University Medical Center, Nashville, TN 37232, United States; Department of Radiation Oncology, Vanderbilt University Medical Center, Nashville, TN 37232, United States; Robert H. Lurie Comprehensive Cancer Center of Northwestern University, Chicago, IL 60611, United States; Division Hematology and Oncology, Vanderbilt-Ingram Cancer Center, Nashville, TN 37232, United States

**Keywords:** anal cancer, squamous cell carcinoma, primary surgery, chemoradiation, SEER, overall survival

## Abstract

**Background:**

Anal cancer incidence is rising in the United States, now exceeding 10 000 cases annually. Chemoradiation (CRT) is the standard curative-intent treatment for non-metastatic squamous cell carcinoma of the anus (SCCA), with surgery generally reserved for non-responders with persistent or progressive disease. Recent trials have refined management, supporting the assessment of response at 26 weeks before considering surgery. However, contemporary population-level patterns of upfront primary surgery and associated survival trends remain incompletely described.

**Methods:**

Using the Surveillance, Epidemiology, and End Results (SEER) registry (2004-2020), we identified adults with newly diagnosed non-metastatic anal squamous cell neoplasms (ICD-O-3 8050-8089). Outcomes were receipt of primary surgery as initial treatment and overall survival. We evaluated temporal trends (Cochran–Armitage), factors associated with primary surgery (multivariable logistic regression), and survival over time (Kaplan–Meier; multivariable Cox proportional hazards modeling).

**Results:**

Among 16 718 patients with non-metastatic anal cancer, 33.1% (*n* = 5529) underwent primary surgery and 83.7% (*n *= 13 997) received radiation as part of initial management. Primary surgery declined from 46.0% in 2004 to 28.7% in 2020 (trend *P* < 0.001), while radiation utilization was relatively stable over time (trend *P* = 0.106). In adjusted analyses, younger age (<50 vs 60-69 years; OR 1.614), male sex (OR 1.508), and Non-Hispanic Black race (vs Non-Hispanic White; OR 1.178) were associated with higher odds of primary surgery. Tumor factors were strongly associated with surgical use (eg, T1 vs T2: OR 3.072; higher N stage associated with lower odds). Overall survival improved across diagnosis periods (log-rank *P* = 0.0002); in adjusted Cox models, diagnosis in 2016-2020 (vs 2004-2007) was associated with lower mortality risk (HR 0.77).

**Conclusions:**

From 2004 to 2020, primary surgery as initial management for non-metastatic anal cancer declined substantially, consistent with increasing adoption of CRT, while overall survival improved over time. Persistent use of upfront surgery in select subgroups warrants further study to clarify indications and ensure guideline-concordant care.

Implications for Practice In this population-based study, primary surgery for non-metastatic anal cancer declined substantially over time, while overall survival improved, supporting the continued shift toward CRT-first, sphincter-preserving management. These findings reinforce the importance of guideline-concordant, multidisciplinary care and careful response assessment before considering surgery. Persistent use of upfront surgery in select subgroups suggests a need for closer evaluation of treatment decision-making, patient counseling, and potential barriers to optimal nonsurgical therapy.

## Introduction

While anal cancer is rare with approximately 11 000 new diagnoses each year in the United States, it is growing in incidence by 2.7% annually with mortality rates increasing 3.1% per year.^1^^,^^2^ Globally, it is estimated to be 50 000 cases per year resulting in 19 000 deaths.^3^ Squamous cell carcinoma of the anus (SCCA) is the most common histology of all anal cancers. It is more commonly diagnosed in women compared to men and Non-Hispanic White, Non-Hispanic Black, and Non-Hispanic American Indian/Alaska Native populations compared to Hispanic and Non-Hispanic Asian/Pacific Islander populations. Median age of diagnosis is 64 years in patients without human immunodeficiency virus (HIV) and <50 years in patients with HIV.^2^^,^^4^ The oncogenic human papillomavirus (HPV), most commonly HPV-16 and HPV-18, are identified in >90% of diagnoses of SCCA.^5^^,^^6^

Majority of SCCA diagnoses are non-metastatic at diagnosis with localized disease being the most common (40%) defined as confined to the primary site followed by regional lymph node involvement (37%), metastatic disease (14%), and unknown staging at diagnosis (8%).^2^ Patients with localized disease also have a higher 5-year survival at 85% compared to those with regional lymph node involvement (70.4%) and distant metastases (36.3%).^2^

Non-metastatic SCCA has a 75-80% cure rate with sphincter preservation when treated with chemoradiation (CRT).^7^^,^^8^ CRT became standard of care following the ACT I trial which showed CRT resulted in fewer locoregional relapses and anal cancer deaths compared to radiation alone.^9^^,^^10^ The phase III ACT II trial and RTOG 98-11 trials established fluorouracil plus mitomycin with 50.4 Gy radiotherapy in 28 daily fractions as the new standard practice. Surgery in non-metastatic SCCA is reserved for cases where the tumor does not respond to CRT or there is progression of disease.

ACT II trial further established the need to wait up until 6 months to allow full response of tumor to CRT due to delayed treatment effect. At 11 weeks after starting treatment, 21% of tumors were still present on exam, but all tumors had a complete response by 26 weeks.^7^

Despite high cure rate results with CRT, surgery is still being utilized upfront though how often is unknown. We investigated the use of upfront surgery in the treatment of non-metastatic SCCA to better understand which patients are not receiving CRT and whether this has changed over time.

## Methods

### Data source

We used Surveillance, Epidemiology, and End Results (SEER) registry data from the National Cancer Institute for years 2004-2020.^11^ SEER provides population-based information on patient demographics, tumor characteristics (site, histology, and TNM stage), initial treatment indicators, and survival outcomes. Because SEER data are de-identified and publicly available, this study was exempt from Institutional Review Board review.

### Study cohort

We identified patients who were newly diagnosed with non-metastatic (ie, M0 according to the TNM staging system) anal cancer characterized by squamous cell neoplasms histology (defined by ICD-O-3 codes ranging from 8050 to 8089) within the SEER database from 2004 to 2020. Patients with TNM staging of T0 or T1N0 were excluded. Because selected T1N0 lesions may appropriately undergo upfront local surgical management, we initially excluded T1N0 cases to focus the primary analysis on patients in whom CRT-first treatment is generally expected. To evaluate the impact of this restriction, we performed a sensitivity analysis reintroducing T1N0 cases into the analytic cohort and repeated the multivariable logistic and Cox regression models.

### Variables of interest

The primary outcomes of interest included the use of primary surgery as an initial treatment strategy and overall survival from diagnosis date. We captured the following patient demographics and clinical characteristics: age at diagnosis (categorized into age groups: <50, 50-59, 60-69, 70-79, 80+ years), sex (Male, Female), race and ethnicity (Non-Hispanic White, Non-Hispanic Black, Hispanic, Non-Hispanic Other), marital status (Married [including common law], Single [never married], Divorced, Separated, Unmarried, Widowed, Unknown), rural-urban status (Counties in metropolitan areas with populations ≥1 million, Counties in metropolitan areas with 250 000 to 1 million population, Counties in metropolitan areas with <250 000 population, Non-metropolitan counties adjacent to a metropolitan area, Non-metropolitan counties not adjacent to a metropolitan area), year of diagnosis (grouped as 2004-2007, 2008-2011, 2012-2015, 2016-2020), T classification (T1, T2, T3, T4, TX) and N classification (N0, N1, N2, N3, NX) of the TNM staging. We categorized anatomic subsite using SEER primary site (ICD-O topography) codes as anal canal [C21.1], overlapping lesion of rectum/anus/anal canal [C21.8], and other anal sites (including anus, not otherwise specified [C21.0] and cloacogenic zone [C21.2]), and included this variable in the multivariable models.

### Statistical analysis

Descriptive statistics were generated for the study cohort. We stratified participants based on whether they underwent primary surgery as their initial treatment and compared demographic and clinical characteristics across groups using chi-squared tests. To explore temporal trends in the adoption of primary surgery, we plotted the proportion of patients who received primary surgery as the initial cancer treatment over the study period. The significance of observed trends was assessed using the Cochrane-Armitage Trend Test. Similarly, we investigated trends in the utilization of radiation therapy through graphical representation and statistical testing.

Multivariable logistic regression analyses were conducted to identify factors influencing the uptake of primary surgery. For survival outcomes, we used Kaplan-Meier estimation to compare survival outcomes by time periods, with the log-rank test applied to detect statistically significant differences. Further, a multivariate Cox proportional hazards model was employed to examine the association between year of diagnosis and overall survival, adjusting for other relevant patient characteristics. We provided hazard ratios (HRs), 95% confidence intervals (CIs), and p-values. Using SEER site-specific surgery codes, we further classified primary surgery into local excision versus abdominoperineal resection (APR) and conducted a sensitivity analysis comparing local excision vs no surgery (excluding APR cases) to assess robustness of the primary surgery findings.

Statistical tests were 2-sided, with results considered statistically significant at *P *< 0.05. All analyses were performed using SAS version 9.4 (SAS Institute Inc., Cary, NC, USA).

## Results

Table 1 provides sample descriptive statistics of this study. The study sample included a total of 16 718 adults diagnosed with nonmetastatic anal cancer from 2004 to 2020. The age distribution of shows a relatively even distribution among the middle-aged groups, with 28.7% and 28.2% of patients being within the 50-59 and 60-69 age ranges, respectively. The older age groups, 70-79 years and 80+ years, comprise a smaller proportion of the cohort, with 16.4% and 10.4%, respectively, while those under 50 years old account for 16.3%. The cohort is predominantly (64.3%) female; the majority of the patients were Non-Hispanic White (78.4%), followed by Non-Hispanic Black (10.0%), and Hispanic (8.8%). The majority of the sample (59.9%) lived in counties within metropolitan areas with populations of 1 million or more. The data reveals an upward trend in the number of cases over the study period, with the number of cases increasing from 582 in year 2004 to 1361 in 2019, before slightly decreasing to 1305 in 2020. Over the whole study period, 33.1% (*n *= 5529) of the patients underwent primary surgery, while 83.7% (*n *= 13 997), received radiation therapy as part of their initial treatment regimen; and 22.91% of the patients received both primary surgery and radiotherapy. Regarding T classification, the majority of patients had T2 tumors (43.8%), followed by TX (27.1%), T3 (16.2%), T4 (9.3%), and T1 (3.7%,). For N classification, most patients were N0 (61.3%), while the remaining patients were distributed across N1 (17.3%), N2 (10.2%), N3 (6.3%), and NX (4.8%). Tumors were most commonly located in the anal canal (46.5%), while overlapping lesions of the rectum, anus, and anal canal accounted for 12.0%.

**Table 1.** Baseline characteristics of patients diagnosed with non-metastatic anal cancer.

Characteristic	Overall (*N* = 16 718)
**Age, n (%)**	
**50-59 years**	4795 (28.7%)
**60-69 years**	4722 (28.2%)
**70-79 years**	2746 (16.4%)
**80+ years**	1735 (10.4%)
**<50 years**	2720 (16.3%)
**Sex, n (%)**	
**Female**	10 748 (64.3%)
**Male**	5970 (35.7%)
**Marital Status, n (%)**	
**Divorced, separated, unmarried**	2767 (16.6%)
**Married (including common law)**	6468 (38.7%)
**Single (never married)**	4558 (27.3%)
**Unknown**	1001 (6.0%)
**Widowed**	1924 (11.5%)
**Race/Ethnicity, n (%)**	
**Hispanic**	1473 (8.8%)
**Non-Hispanic White**	13 111 (78.4%)
**Non-Hispanic Black**	1668 (10.0%)
**Non-Hispanic Other**	466 (2.8%)
**Year of diagnosis, n (%)**	
**2004**	582 (3.5%)
**2005**	686 (4.1%)
**2006**	650 (3.9%)
**2007**	756 (4.5%)
**2008**	824 (4.9%)
**2009**	888 (5.3%)
**2010**	914 (5.5%)
**2011**	887 (5.3%)
**2012**	963 (5.8%)
**2013**	1026 (6.1%)
**2014**	1086 (6.5%)
**2015**	1137 (6.8%)
**2016**	1167 (7.0%)
**2017**	1158 (6.9%)
**2018**	1328 (7.9%)
**2019**	1361 (8.1%)
**2020**	1305 (7.8%)
**Primary surgery, n (%)**	
**Yes**	5529 (33.1%)
**No**	11 189 (66.9%)
**Radiation, n (%)**	
**No**	2721 (16.3%)
**Yes**	13 997 (83.7%)
**Rural-Urban Continuum Code, n (%)**	
**Metro counties, >1 million population**	10 010 (59.9%)
**Metro counties, 250** **000-1 million population**	3422 (20.5%)
**Metro counties, <250** **000 population**	1308 (7.8%)
**Nonmetro, adjacent to metro area**	1173 (7.0%)
**Nonmetro, not adjacent to metro area**	805 (4.8%)
**T classification, n (%)**	
**T1**	611 (3.7%)
**T2**	7317 (43.8%)
**T3**	2703 (16.2%)
**T4**	1558 (9.3%)
**TX**	4529 (27.1%)
**N classification, n (%)**	
**N0**	10 253 (61.3%)
**N1**	2899 (17.3%)
**N2**	1705 (10.2%)
**N3**	1055 (6.3%)
**NX**	806 (4.8%)
**Subsite category, n (%)**	
**Anus, NOS or cloacogenic zone**	6928 (41.4%)
**Anal canal**	7780 (46.5%)
**Overlapping lesion of rectum, anus, and anal canal**	2010 (12.0%)

**Figure 1.**
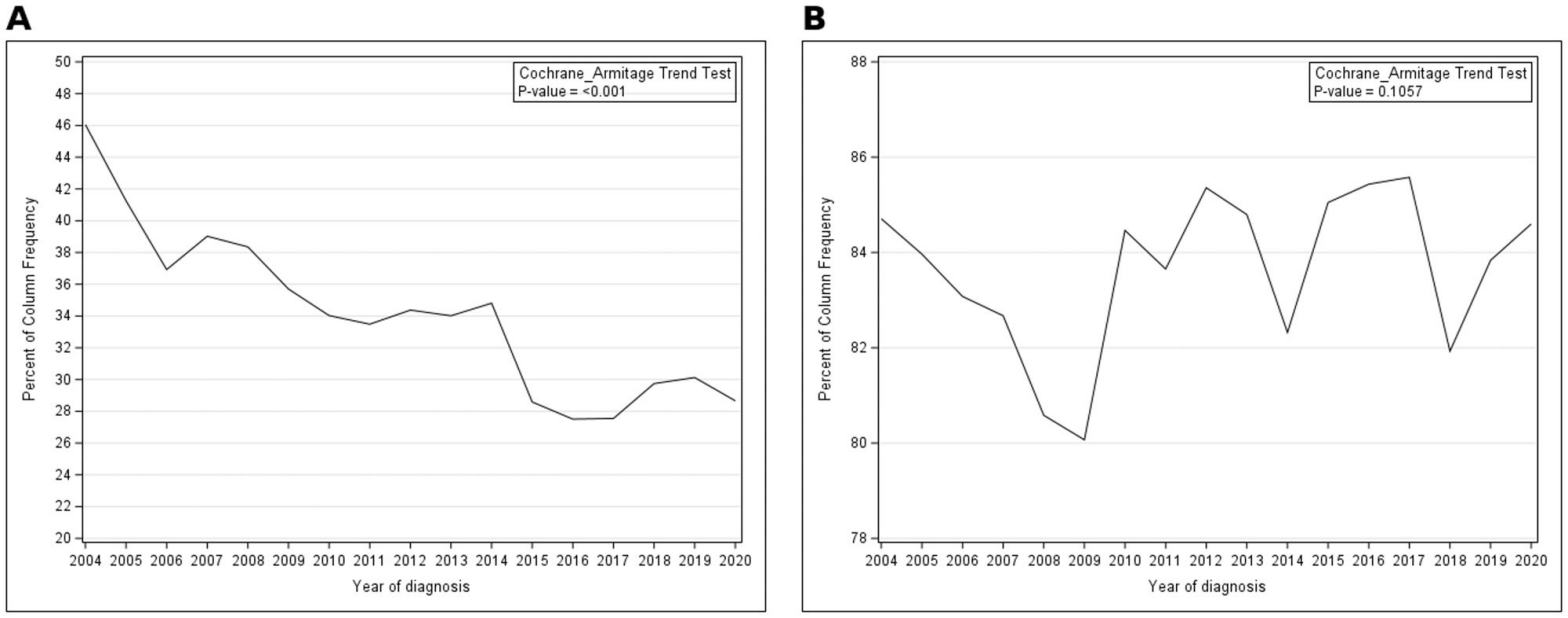
(A) Temporal trends in primary surgery (2004-2020). Annual proportion of patients who underwent primary surgery as part of initial management, stratified by year of diagnosis (SEER 2004-2020). (B) Temporal trends in radiotherapy use (2004-2020). Annual proportion of patients who received radiotherapy as part of initial management, stratified by year of diagnosis (SEER 2004-2020).

Table 2 shows the use of primary surgery by different subgroups of patients. The chi-square tests demonstrated that the use of primary surgery varies significantly by age, sex, race/ethnicity, marital status, year of diagnosis, T and N classifications. Younger, male, non-Hispanic Black patients were significantly more likely to have surgery (*P* < 0.001). The use of primary surgery has been declining over the study period from 46.0% in 2004 to 28.7% in 2020. Figure 1A illustrates the proportion of patients with primary surgery over the years from 2004 to 2020. We observed a significant decrease in the use of primary surgery during this study period with the Cochrane Armitage trend test showing a p-value of less than 0.001. In contrast, during the same time period, the use of radiation therapy was relatively stable with the Cochrane Armitage trend test showing a p-value of 0.106 (Figure 1B).

**Table 2.** Patient characteristics by receipt of primary surgery.

Characteristic	Primary surgery: Yes	Primary surgery: No	*P* value
**Age, n (%)**			<.0001
**<50 years**	1575 (32.8%)	3220 (67.2%)	
**50-59 years**	1421 (30.1%)	3301 (69.9%)	
**60-69 years**	835 (30.4%)	1911 (69.6%)	
**70-79 years**	510 (29.4%)	1225 (70.6%)	
**80+ years**	1188 (43.7%)	1532 (56.3%)	
**Sex, n (%)**			<.0001
**Female**	3107 (28.9%)	7641 (71.1%)	
**Male**	2422 (40.6%)	3548 (59.4%)	
**Marital Status, n (%)**			<.0001
**Divorced, separated, unmarried**	812 (29.3%)	1955 (70.7%)	
**Married (including common law)**	2151 (33.3%)	4317 (66.7%)	
**Single (never married)**	1701 (37.3%)	2857 (62.7%)	
**Unknown**	329 (32.9%)	672 (67.1%)	
**Widowed**	536 (27.9%)	1388 (72.1%)	
**Race/Ethnicity, n (%)**			<.0001
**Hispanic**	499 (33.9%)	974 (66.1%)	
**Non-Hispanic White**	4232 (32.3%)	8879 (67.7%)	
**Non-Hispanic Black**	639 (38.3%)	1029 (61.7%)	
**Non-Hispanic Other**	159 (34.1%)	307 (65.9%)	
**Year of diagnosis, n (%)**			<.0001
**2004**	268 (46.0%)	314 (54.0%)	
**2005**	283 (41.3%)	403 (58.7%)	
**2006**	240 (36.9%)	410 (63.1%)	
**2007**	295 (39.0%)	461 (61.0%)	
**2008**	316 (38.3%)	508 (61.7%)	
**2009**	317 (35.7%)	571 (64.3%)	
**2010**	311 (34.0%)	603 (66.0%)	
**2011**	297 (33.5%)	590 (66.5%)	
**2012**	331 (34.4%)	632 (65.6%)	
**2013**	349 (34.0%)	677 (66.0%)	
**2014**	378 (34.8%)	708 (65.2%)	
**2015**	325 (28.6%)	812 (71.4%)	
**2016**	321 (27.5%)	846 (72.5%)	
**2017**	319 (27.5%)	839 (72.5%)	
**2018**	395 (29.7%)	933 (70.3%)	
**2019**	410 (30.1%)	951 (69.9%)	
**2020**	374 (28.7%)	931 (71.3%)	
**Radiation, n (%)**			<.0001
**No**	1697 (62.4%)	1024 (37.6%)	
**Yes**	3832 (27.4%)	10 165 (72.6%)	
**Rural-Urban Continuum Code, n (%)**			0.33721
**Metro counties, >1 million population**	3353 (33.5%)	6657 (66.5%)	
**Metro counties, 250** **000-1 million population**	1107 (32.3%)	2315 (67.7%)	
**Metro counties, <250** **000 population**	431 (33.0%)	877 (67.0%)	
**Nonmetro, adjacent to metro area**	394 (33.6%)	779 (66.4%)	
**Nonmetro, not adjacent to metro area**	244 (30.3%)	561 (69.7%)	
**T classification, n (%)**			<.0001
**T1**	293 (48.0%)	318 (52.0%)	
**T2**	2483 (33.9%)	4834 (66.1%)	
**T3**	646 (23.9%)	2057 (76.1%)	
**T4**	319 (20.5%)	1239 (79.5%)	
**TX**	1788 (39.5%)	2741 (60.5%)	
**N classification, n (%)**			<.0001
**N0**	3977 (38.8%)	6276 (61.2%)	
**N1**	639 (22.0%)	2260 (78.0%)	
**N2**	426 (25.0%)	1279 (75.0%)	
**N3**	183 (17.3%)	872 (82.7%)	
**NX**	304 (37.7%)	502 (62.3%)	
**Subsite category, n (%)**			<.0001
**Anus, NOS or cloacogenic zone**	2554 (36.9%)	4374 (63.1%)	
**Anal canal**	2328 (29.9%)	5452 (70.1%)	
**Overlapping lesion of rectum, anus, and anal canal**	647 (32.2%)	1363 (67.8%)	

The results from the multivariable logistic regression for the use of primary surgery are provided in Table 3. It shows that younger patients under 50 years exhibited a notably higher likelihood of undergoing primary surgery (Odds Ratio [OR] = 1.614, *P* < .0001) compared to the reference group (60-69 years). Males were also more likely to receive surgery than females (OR = 1.508, *P* < .0001), and non-Hispanic Blacks were more likely to undergo surgery compared to Non-Hispanic Whites (OR = 1.178, *P* = 0.005). Tumor classification also showed significant impact on the use of surgery, with T1 tumors being more than 3 times as likely to be managed surgically compared to T2 tumors (OR = 3.072, *P* < .0001), while T3 and T4 were associated with lower surgery odds (OR = 0.682 and OR = 0.637, respectively, both *P* < .0001). Similarly, patients with higher N classifications demonstrated significantly reduced odds of undergoing surgery (eg, OR = 0.319, *P* < .0001 for N3 status compared to N0), indicating less surgical intervention as nodal involvement increases. Controlling for all other factors, we still observe a significant downward trend in surgery uptake over the years, with the odds significantly lower in the later periods (eg, OR = 0.720, *P* < .0001 for 2016-2020) relative to the earliest period (2004-2007). Finally, anatomic subsite was also associated with primary surgery; compared with anal canal tumors, surgery was more common for overlapping anorectal lesions (OR = 1.188; *P* = 0.002).

**Table 3.** Multivariable logistic regression for receipt of primary surgery.

Parameter	OR	95% CI	*P* value	Sig
**Age**				
**50-59 years**	1.089	(1.00, 1.19)	0.0640	
**70-79 years**	1.014	(0.91, 1.13)	0.7958	
**80+ years**	0.923	(0.81, 1.05)	0.2351	
**<50 years**	1.614	(1.45, 1.80)	<.0001	***
**60-69 years**	Reference			
**Sex**				
**Male**	1.508	(1.40, 1.62)	<.0001	***
**Female**	Reference			
**Marital Status**				
**Divorced, separated, unmarried**	0.861	(0.78, 0.95)	0.0036	**
**Single (never married)**	0.965	(0.88, 1.05)	0.4335	
**Unknown**	0.853	(0.73, 0.99)	0.0368	*
**Widowed**	0.880	(0.78, 1.00)	0.0456	*
**Married (including common law)**	Reference			
**Race/Ethnicity**				
**Hispanic**	1.035	(0.92, 1.17)	0.5736	
**Non-Hispanic Black**	1.178	(1.05, 1.32)	0.0050	**
**Non-Hispanic Other**	1.125	(0.92, 1.38)	0.2569	
**Non-Hispanic White**	Reference			
**Year of Diagnosis**				
**2008-2011**	0.855	(0.77, 0.95)	0.0046	**
**2012-2015**	0.842	(0.76, 0.94)	0.0015	**
**2016-2020**	0.720	(0.65, 0.80)	<.0001	***
**2004-2007**	Reference			
**Rural-Urban Continuum Code**				
**Metro counties, 250** **000-1 million population**	1.014	(0.93, 1.11)	0.7528	
**Metro counties, <250** **000 population**	1.022	(0.90, 1.16)	0.7325	
**Nonmetro, adjacent to metro area**	1.073	(0.94, 1.23)	0.3022	
**Nonmetro, not adjacent to metro area**	0.869	(0.74, 1.02)	0.0911	
**Metro counties, >1 million population**	Reference			
**T Classification**				
**T1**	3.072	(2.57, 3.68)	<.0001	***
**T3**	0.682	(0.61, 0.76)	<.0001	***
**T4**	0.637	(0.56, 0.73)	<.0001	***
**TX**	1.101	(1.02, 1.20)	0.0199	*
**T2**	Reference			
**N Classification**				
**N1**	0.454	(0.41, 0.51)	<.0001	***
**N2**	0.462	(0.41, 0.52)	<.0001	***
**N3**	0.319	(0.27, 0.38)	<.0001	***
**NX**	0.787	(0.67, 0.92)	0.0032	**
**N0**	Reference			
**Subsite Category**				
**Anus, NOS or cloacogenic zone**	1.298	(1.21, 1.40)	<.0001	***
**Overlapping lesion of rectum, anus, and anal canal**	1.188	(1.07, 1.33)	0.0020	**
**Anal canal**	Reference			

**Figure 2.**
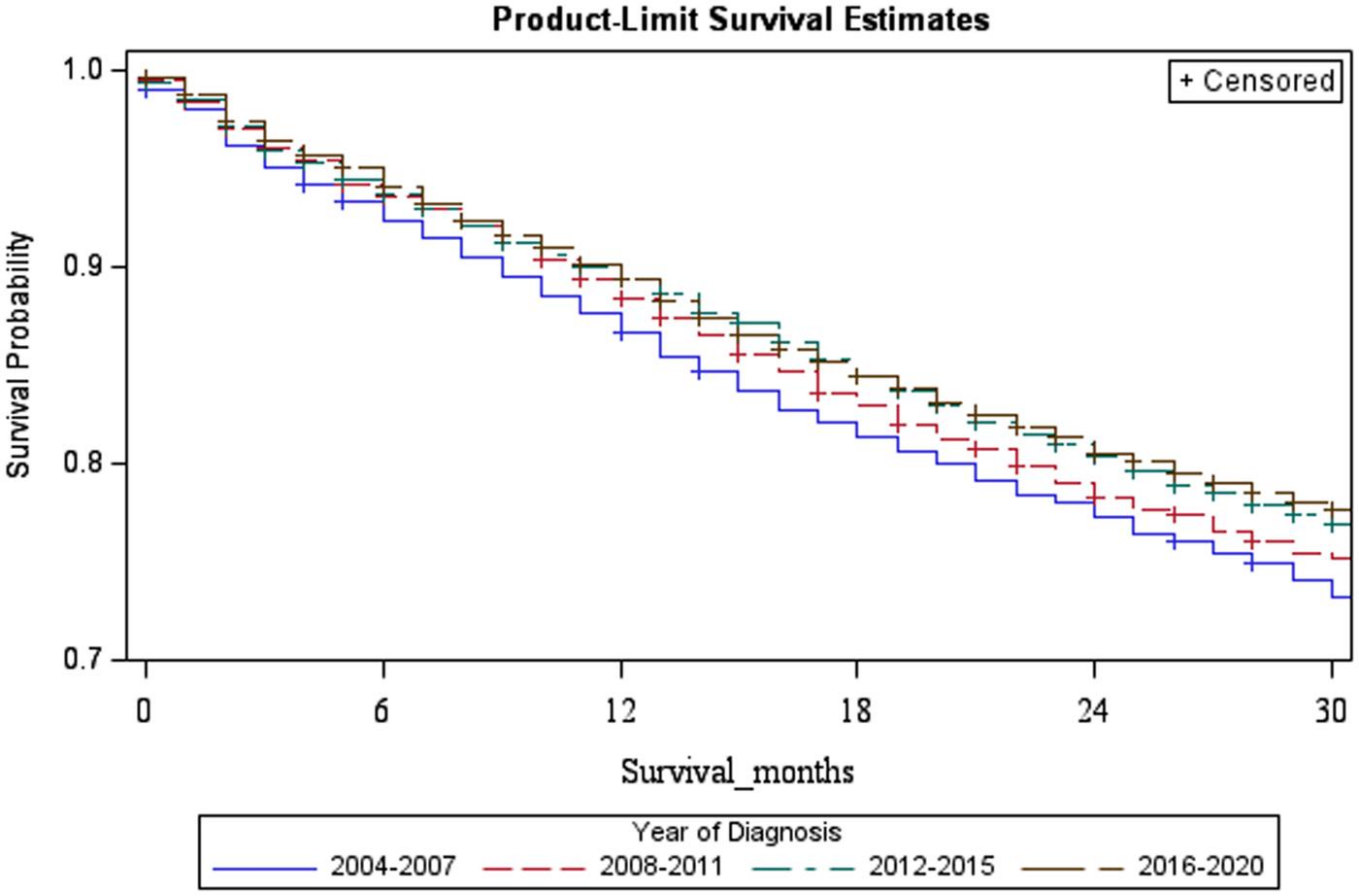
Overall survival by calendar period of diagnosis. Kaplan–Meier overall survival curves for patients diagnosed in 2004-2007, 2008-2011, 2012-2015, and 2016-2020 (SEER 2004-2020). Differences across curves were assessed using the log-rank test.

In terms of overall survival outcomes, the Kaplan-Meier curves describing the survival trends in the years 2004-2007, 2008-2011, 2012-2015, 2016-2020 shows that the overall survival has been improving over these time periods significantly with a log rank test showing p-value of 0.0002 (Figure 2). The results from the multivariable Cox proportional hazard model are presented in Table 4. We found that age was significantly associated with survival outcomes as expected with younger patients (50-59 years) having a lower risk of mortality (Hazard Ratio [HR] = 0.78, *P* < 0.001), while older age groups (70-79 years, HR = 1.65; 80+ years, HR = 3.23, both *P* < 0.001) having significantly increased risks compared to the 60-69 years-old reference group. Males had a higher risk compared to females (HR = 1.56, *P* < 0.001). We also found that non-Hispanic Blacks had a higher risk of mortality compared to Non-Hispanic Whites (HR = 1.25, *P* < 0.001), while Non-Hispanic Others had a lower risk (HR = 0.73, *P* < 0.001). As expected, tumor staging was a strong predictor of outcomes. Patients with T1 tumors had a decreased risk (HR = 0.74, *P* < 0.001), while those with higher T stages (T3 and T4) faced increased risks (HR = 1.45 and 1.74 respectively, *P* < 0.001). Similarly, advancing N classification was associated with increased mortality, highlighting the prognostic significance of regional lymph node involvement in anal cancer. Regarding the year of diagnosis, patients diagnosed more recently (2016-2020) demonstrated a significantly lower risk of mortality compared to those diagnosed in 2004-2007 (HR = 0.77, *P* < 0.001). Subsite was independently associated with survival, with higher mortality for overlapping anorectal lesions (HR = 1.17, *P* < 0.001) compared with anal canal tumors.

**Table 4.** Multivariable Cox proportional hazards model for overall survival.

Parameter	HR	95% CI	*P* value	Sig
**Age categories**				
**60-69 years**	Reference			
**50-59 years**	0.78	[0.73, 0.84]	<0.001	***
**70-79 years**	1.65	[1.52, 1.78]	<0.001	***
**80+ years**	3.23	[2.98, 3.51]	<0.001	***
**<50 years**	0.66	[0.61, 0.72]	<0.001	***
**Sex**				
**Female**	Reference			
**Male**	1.56	[1.48, 1.65]	<0.001	***
**Marital status**				
**Married (including common law)**	Reference			
**Divorced, separated, unmarried**	1.33	[1.23, 1.43]	<0.001	***
**Single (never married)**	1.37	[1.28, 1.46]	<0.001	***
**Unknown**	1.06	[0.94, 1.20]	0.316	
**Widowed**	1.48	[1.37, 1.60]	<0.001	***
**Race/Ethnicity**				
**Non-Hispanic White**	Reference			
**Hispanic**	0.98	[0.90, 1.08]	0.718	
**Non-Hispanic Black**	1.25	[1.15, 1.36]	<0.001	***
**Non-Hispanic Other**	0.73	[0.61, 0.87]	<0.001	***
**Year of diagnosis**				
**2004-2007**	Reference			
**2008-2011**	0.94	[0.88, 1.01]	0.086	
**2012-2015**	0.87	[0.81, 0.94]	<0.001	***
**2016-2020**	0.77	[0.71, 0.84]	<0.001	***
**Rural-urban status**				
**Metro counties, >1 million population**	Reference			
**Metro counties, 250** **000-1 million population**	1.11	[1.04, 1.18]	0.002	**
**Metro counties, <250** **000 population**	1.20	[1.09, 1.31]	<0.001	***
**Nonmetro, adjacent to metro area**	1.17	[1.06, 1.29]	0.002	**
**Nonmetro, not adjacent to metro area**	1.22	[1.09, 1.37]	0.001	**
**T classification**				
**T2**	Reference			
**T1**	0.74	[0.62, 0.87]	<0.001	***
**T3**	1.45	[1.35, 1.56]	<0.001	***
**T4**	1.74	[1.60, 1.90]	<0.001	***
**TX**	1.17	[1.10, 1.24]	<0.001	***
**N classification**				
**N0**	Reference			
**N1**	1.13	[1.04, 1.23]	0.003	**
**N2**	1.23	[1.14, 1.34]	<0.001	***
**N3**	1.50	[1.37, 1.65]	<0.001	***
**NX**	1.43	[1.27, 1.61]	<0.001	***
**Subsite category**				
**Anal canal**	Reference			
**Anus, NOS or cloacogenic zone**	1.09	[1.03, 1.15]	0.002	**
**Overlapping lesion of rectum, anus, and anal canal**	1.17	[1.09, 1.27]	<0.001	***

* *P < *0.05;

** *P < *0.01;

*** *P < *0.001.

In the sensitivity analysis reintroducing T1N0 cases, the main findings were materially unchanged. The temporal decline in primary surgery persisted (2016-2020 vs 2004-2007: OR, 0.743; 95% CI, 0.68-0.81), and the survival model remained consistent, with more recent diagnosis associated with improved survival (2016-2020 vs 2004-2007: HR 0.78, 95% CI 0.72-0.84).

Among patients undergoing primary surgery, the vast majority underwent local excision (*n* = 4664), whereas APR was uncommon (*n* = 716). In sensitivity analyses restricted to local excision vs no surgery, the associations and temporal trends were essentially unchanged. Detailed results on these sensitivity analyses are provided in the Supplementary Materials.

## Discussion

This retrospective analysis of SEER registry data evaluated which patient populations are still undergoing primary surgery and the frequency at which primary surgery has been used over time for non-metastatic anal cancer during a 16-year period (2004-2020).

The patient population consisted of mostly patients aged 50 to 69 (56.9%), predominantly female (64.3%), and majority were Non-Hispanic White (78.4%), Non-Hispanic Black (10.0%), and Hispanic (8.8%) which is consistent with prior demographics of this population reported.

The study also found an increasing number of cases of anal cancer being diagnosed. Despite this finding, overall survival was noted to be improving as well with younger patients having lower risk of mortality (HR = 0.78, *P* < 0.001) but male patients (HR = 1.56, *P* < 0.001) and non-Hispanic Black patients (HR =1.25, *P* < 0.001) having higher risk of mortality. Risk of mortality was also slightly higher in patients living in less populated non-metropolitan (HR = 1.20) areas and smaller metropolitan areas (HR = 1.17) compared to large metropolitan areas (HR =1.23, *P* ≤ 0.001).

Most patients who received surgery had T2 tumors (43.8%) and were N0 (61.3%). Tumor staging had prognostic implications. Similar to historical data from RTOG 98-11,^7^ higher staging of T and N both were associated with worse overall survival.

Our finding that primary surgery declined substantially over time is directionally consistent with the evolution of evidence-based anal cancer management. Randomized phase III trials established CRT as the standard curative-intent treatment for non-metastatic SCCA and supported reserving radical surgery for salvage. The UKCCCR trial demonstrated improved outcomes with radiotherapy combined with 5-fluorouracil and mitomycin compared with radiotherapy alone, supporting CRT as standard and surgery for failures.^9^^,^^10^ Subsequent trials refined CRT regimens and long-term outcomes (eg, RTOG 98-11), but did not re-establish upfront surgery as preferred for most non-metastatic disease.^7^ Importantly, post-hoc analyses from ACT II indicate that complete clinical response may occur later than early post-treatment assessments and support response evaluation at approximately 26 weeks before committing patients to salvage surgery.^7^^,^^8^ Taken together, these trial results provide a strong rationale for the declining use of primary surgery observed in our real-world cohort.

Building on this trial foundation, contemporaneous guideline development and increasing multidisciplinary coordination likely reinforced the shift away from primary surgery during the study period. Throughout the study period, major national and international guidelines (eg, NCCN and ESMO/ESSO/ESTRO) consistently endorsed concurrent chemoradiotherapy as the standard initial management for most stage I-III anal canal cancers, reserving abdominoperineal resection for persistent or recurrent disease and limiting local excision to carefully selected early anal margin or superficially invasive lesions with favorable features. Particularly in the later portion of the study period, guidelines increasingly emphasized structured response assessment and avoidance of premature salvage surgery, acknowledging that complete clinical response may occur several months following CRT. In parallel, care delivery increasingly incorporated multidisciplinary tumor board–driven management. This coordinated approach together with broader adoption of modern conformal radiotherapy techniques and improvements in supportive care may have facilitated greater adherence to CRT-first strategies and plausibly contributed to both the observed decline in primary surgery and improvements in overall survival at the population level.

Despite the high cure rates with treatment using CRT, primary surgery is still being performed. The rate of primary surgery was found to decline during this study period from 46.6% in 2004 to 31.1% in 2020. One of the limitations of this study is not knowing the type of surgery performed. Abdominoperineal resection is the preferred surgery following progression or recurrence after CRT.^12–15^ However, there are instances prior to CRT that a fecal diversion may be done such as development of fistulas or obstruction.

Although exclusion of T1N0 disease was intended to avoid conflating an accepted early-stage surgical subgroup with the broader CRT-first population, sensitivity analyses that reintroduced T1N0 cases showed no material change in the observed temporal trends or multivariable associations, supporting the stability of the main results.

Another limitation of this study is not knowing the reason behind why primary surgery was done. Decision for primary surgery may have been driven by medical team or patient preference, for example. SEER does not capture treatment intent, nor does it include key radiotherapy parameters such as dose, fractionation, target volumes, treatment breaks, or the detailed timing of treatment initiation and completion. Chemotherapy use and regimen details are also not reliably available, limiting the ability to distinguish omission from delay of CRT or to assess whether outcomes are driven by treatment completion and timeliness. Furthermore, while SEER’s radiotherapy modality variable indicated that nearly all recorded radiotherapy was beam radiation (98.51%), with very few implant/isotope cases; SEER does not identify major within-category advances in external beam radiotherapy delivery during the study period such as broader adoption of CT-based 3-dimensional conformal techniques, intensity-modulated radiotherapy/volumetric-modulated arc therapy, and image guidance which may influence toxicity, treatment tolerance, and ultimately survival outcomes. Consequently, survival trends observed over calendar time likely reflect a combination of secular improvements in radiotherapy delivery, systemic therapy, supportive care, and survivorship management, in addition to evolving adherence to CRT-first treatment paradigms, and should not be interpreted as causal comparative effectiveness estimates.

Some patients may have reservations about having chemotherapy or radiation and prefer surgery. Younger, male, non-Hispanic patients were found to have higher rates of primary surgery (*P* < 0.001) using chi-square tests in the study. This suggests that there could be concerns such as premature ovarian dysfunction or infertility that drove the decision in this population. The multivariable logistic regression noted patients under 50 years were more likely to undergo primary surgery as well (OR = 1.614, *P* < 0.0001) compared to patients ages 60 to 69 years. Some have advocated for upfront surgery of anal cancers in patients with Crohn’s disease which may have contributed to the percent of patients undergoing primary surgery.^16^^,^^17^ Additionally, human immunodeficiency virus (HIV) status was not captured in this study, so it is unclear if HIV status may have played a role in decision for primary surgery.

SEER does not capture disease progression or recurrence (including locoregional failure or distant metastasis after diagnosis), precluding evaluation of locoregional control, metastasis-free survival, or recurrence-free survival; accordingly, overall survival was the primary longitudinal outcome available for this cohort. Future randomized trials and studies linking registry data to more granular clinical sources (eg, institutional EHR/oncology records, claims data, or prospective cooperative-group datasets) are needed to characterize recurrence patterns and disease-control endpoints and to distinguish improvements in locoregional versus distant control over time.

Regardless, the annual incidence of anal cancer is rising with increasing rates of overall survival. While there are still some patients undergoing primary surgery for non-metastatic anal cancer. Overall, the number of patients has decreased over the past 2 decades signaling the increased adoption of CRT as the primary treatment modality before considering surgery. Our findings underscore the positive impact of modern phase III clinical trials on shaping the treatment paradigm of a rare cancer enabling sphincter preservation with curative intent. Historically, pharmaceutical companies have been reluctant to pursue large clinical trials in a malignancy considered rare. Consequently, within the last decade, the National Cancer Institute’s National Clinical Trials Network (NCTN) has emerged as the primary sponsor for 3 pivotal phase III trials: EA2165 (NCT03233711), EA2182 (NT04166318) and EA2176 (044444921); each addressing critical questions about the current treatment paradigm. All 3 trials have completed enrollment; results are pending. Hence given the continued rising incidence of squamous cell carcinoma of the anal canal, we hope these findings will stimulate ongoing investigation and the pursuit of novel therapeutic approaches.

## Supplementary Material

oyag148_Supplementary_Data

## Data Availability

The data underlying this article were accessed from the National Cancer Institute’s Surveillance, Epidemiology, and End Results (SEER) Program. SEER research data are publicly available to qualified investigators through the SEER Program following completion of the applicable SEER Research Data Agreement.
