# Supplementary material for: Trends in primary surgery and overall survival in non-metastatic anal cancer: a population-based analysis
Source: Oncologist. 2026 Apr 17;31(6):oyag148. doi: 10.1093/oncolo/oyag148 (PMC13153677; doi:10.1093/oncolo/oyag148)
Supplement: oyag148_Supplementary_Data [file oyag148_supplementary_data.docx]

**Supplementary Materials**

**Supplementary Analysis A. Reintroduction of T1N0 cases into the analytic cohort**

This section reports the sensitivity analysis in which T1N0 cases were added back into the cohort and the multivariable models were re-estimated.

**Supplementary Table S1. Cohort characteristics for the sensitivity analysis including T1N0 cases (N=20,289).**

| **Characteristic** | **Value** |
| --- | --- |
| **Age, n (%)** | |
| 50-59 years | 5839 (28.8%) |
| 60-69 years | 5760 (28.4%) |
| 70-79 years | 3363 (16.6%) |
| 80+ years | 2019 (10.0%) |
| <50 years | 3308 (16.3%) |
| **Sex, n (%)** | |
| Female | 12984 (64.0%) |
| Male | 7305 (36.0%) |
| **Marital status, n (%)** | |
| Divorced, separated, unmarried | 3301 (16.3%) |
| Married (including common law) | 8011 (39.5%) |
| Single (never married) | 5498 (27.1%) |
| Unknown | 1190 (5.9%) |
| Widowed | 2289 (11.3%) |
| **Race/ethnicity, n (%)** | |
| Hispanic | 1791 (8.8%) |
| Non-Hispanic White | 15970 (78.7%) |
| Non-Hispanic Black | 1973 (9.7%) |
| Non-Hispanic Other | 555 (2.7%) |
| **Year of diagnosis, n (%)** | |
| 2004 | 718 (3.5%) |
| 2005 | 846 (4.2%) |
| 2006 | 797 (3.9%) |
| 2007 | 888 (4.4%) |
| 2008 | 998 (4.9%) |
| 2009 | 1085 (5.3%) |
| 2010 | 1126 (5.5%) |
| 2011 | 1119 (5.5%) |
| 2012 | 1199 (5.9%) |
| 2013 | 1271 (6.3%) |
| 2014 | 1304 (6.4%) |
| 2015 | 1399 (6.9%) |
| 2016 | 1453 (7.2%) |
| 2017 | 1406 (6.9%) |
| 2018 | 1548 (7.6%) |
| 2019 | 1599 (7.9%) |
| 2020 | 1533 (7.6%) |
| **Primary surgery, n (%)** | |
| Yes | 7820 (38.5%) |
| No | 12469 (61.5%) |
| **Radiation, n (%)** | |
| Yes | 16439 (81.0%) |
| No | 3850 (19.0%) |
| **Rural-urban continuum code, n (%)** | |
| Metro >=1 million | 12244 (60.3%) |
| Metro 250,000 to 1 million | 4105 (20.2%) |
| Metro <250,000 | 1543 (7.6%) |
| Nonmetropolitan adjacent | 1418 (7.0%) |
| Nonmetropolitan nonadjacent | 979 (4.8%) |
| **T classification, n (%)** | |
| T1 | 4182 (20.6%) |
| T2 | 7317 (36.1%) |
| T3 | 2703 (13.3%) |
| T4 | 1558 (7.7%) |
| TX | 4529 (22.3%) |
| **N classification, n (%)** | |
| N0 | 13824 (68.1%) |
| N1 | 2899 (14.3%) |
| N2 | 1705 (8.4%) |
| N3 | 1055 (5.2%) |
| NX | 806 (4.0%) |
| **Subsite category, n (%)** | |
| Anus, NOS or cloacogenic zone | 8436 (41.6%) |
| Anal canal | 9475 (46.7%) |
| Overlapping lesion of rectum, anus, and anal canal | 2378 (11.7%) |

**Supplementary Table S2. Patient characteristics by receipt of primary surgery in the sensitivity analysis including T1N0 cases.**

| **Characteristic** | **Level** | **Yes** | **No** | **P value** |
| --- | --- | --- | --- | --- |
| **Age, n (%)** | | | | |
|  | 50-59 years | 2269 (38.9%) | 3570 (61.1%) | <.0001 |
|  | 60-69 years | 2058 (35.7%) | 3702 (64.3%) |  |
|  | 70-79 years | 1209 (36.0%) | 2154 (64.0%) |  |
|  | 80+ years | 671 (33.2%) | 1348 (66.8%) |  |
|  | <50 years | 1613 (48.8%) | 1695 (51.2%) |  |
| **Sex, n (%)** | | | | |
|  | Female | 4435 (34.2%) | 8549 (65.8%) | <.0001 |
|  | Male | 3385 (46.3%) | 3920 (53.7%) |  |
| **Marital status, n (%)** | | | | |
|  | Divorced, separated, unmarried | 1144 (34.7%) | 2157 (65.3%) | <.0001 |
|  | Married (including common law) | 3093 (38.6%) | 4918 (61.4%) |  |
|  | Single (never married) | 2385 (43.4%) | 3113 (56.6%) |  |
|  | Unknown | 449 (37.7%) | 741 (62.3%) |  |
|  | Widowed | 749 (32.7%) | 1540 (67.3%) |  |
| **Race/ethnicity, n (%)** | | | | |
|  | Hispanic | 702 (39.2%) | 1089 (60.8%) | <.0001 |
|  | Non-Hispanic White | 6039 (37.8%) | 9931 (62.2%) |  |
|  | Non-Hispanic Black | 868 (44.0%) | 1105 (56.0%) |  |
|  | Non-Hispanic Other | 211 (38.0%) | 344 (62.0%) |  |
| **Year of diagnosis, n (%)** | | | | |
|  | 2004 | 358 (49.9%) | 360 (50.1%) | <.0001 |
|  | 2005 | 389 (46.0%) | 457 (54.0%) |  |
|  | 2006 | 338 (42.4%) | 459 (57.6%) |  |
|  | 2007 | 383 (43.1%) | 505 (56.9%) |  |
|  | 2008 | 432 (43.3%) | 566 (56.7%) |  |
|  | 2009 | 445 (41.0%) | 640 (59.0%) |  |
|  | 2010 | 446 (39.6%) | 680 (60.4%) |  |
|  | 2011 | 454 (40.6%) | 665 (59.4%) |  |
|  | 2012 | 498 (41.5%) | 701 (58.5%) |  |
|  | 2013 | 501 (39.4%) | 770 (60.6%) |  |
|  | 2014 | 513 (39.3%) | 791 (60.7%) |  |
|  | 2015 | 489 (35.0%) | 910 (65.0%) |  |
|  | 2016 | 509 (35.0%) | 944 (65.0%) |  |
|  | 2017 | 470 (33.4%) | 936 (66.6%) |  |
|  | 2018 | 531 (34.3%) | 1017 (65.7%) |  |
|  | 2019 | 561 (35.1%) | 1038 (64.9%) |  |
|  | 2020 | 503 (32.8%) | 1030 (67.2%) |  |
| **Radiation, n (%)** | | | | |
|  | No | 2734 (71.0%) | 1116 (29.0%) | <.0001 |
|  | Yes | 5086 (30.9%) | 11353 (69.1%) |  |
| **Rural-urban continuum code, n (%)** | | | | |
|  | Metro >=1 million | 4797 (39.2%) | 7447 (60.8%) | 0.1222 |
|  | Metro 250,000 to 1 million | 1544 (37.6%) | 2561 (62.4%) |  |
|  | Metro <250,000 | 579 (37.5%) | 964 (62.5%) |  |
|  | Nonmetropolitan adjacent | 549 (38.7%) | 869 (61.3%) |  |
|  | Nonmetropolitan nonadjacent | 351 (35.9%) | 628 (64.1%) |  |
| **T classification, n (%)** | | | | |
|  | T1 | 2584 (61.8%) | 1598 (38.2%) | <.0001 |
|  | T2 | 2483 (33.9%) | 4834 (66.1%) |  |
|  | T3 | 646 (23.9%) | 2057 (76.1%) |  |
|  | T4 | 319 (20.5%) | 1239 (79.5%) |  |
|  | TX | 1788 (39.5%) | 2741 (60.5%) |  |
| **N classification, n (%)** | | | | |
|  | N0 | 6268 (45.3%) | 7556 (54.7%) | <.0001 |
|  | N1 | 639 (22.0%) | 2260 (78.0%) |  |
|  | N2 | 426 (25.0%) | 1279 (75.0%) |  |
|  | N3 | 183 (17.3%) | 872 (82.7%) |  |
|  | NX | 304 (37.7%) | 502 (62.3%) |  |
| **Subsite category, n (%)** | | | | |
|  | Anus, NOS or cloacogenic zone | 3643 (43.2%) | 4793 (56.8%) | <.0001 |
|  | Anal canal | 3304 (34.9%) | 6171 (65.1%) |  |
|  | Overlapping lesion of rectum, anus, and anal canal | 873 (36.7%) | 1505 (63.3%) |  |

*Abbreviation: APR, abdominoperineal resection. P values from chi-square tests.*

**Supplementary Table S3. Multivariable logistic regression for receipt of primary surgery in the sensitivity analysis including T1N0 cases.**

| **Factor** | **Level** | **Odds ratio** | **95% CI** | **P value** | **Sig** |
| --- | --- | --- | --- | --- | --- |
| **Age** | | | | | |
|  | 50-59 years | 1.101 | (1.02, 1.19) | 0.0188 | * |
|  | 70-79 years | 1.008 | (0.92, 1.11) | 0.8609 |  |
|  | 80+ years | 0.903 | (0.80, 1.02) | 0.0939 |  |
|  | <50 years | 1.549 | (1.41, 1.71) | <.0001 | *** |
|  | 60-69 years | Reference |  |  |  |
| **Sex** | | | | | |
|  | Male | 1.509 | (1.41, 1.61) | <.0001 | *** |
|  | Female | Reference |  |  |  |
| **Marital status** | | | | | |
|  | Divorced, separated, unmarried | 0.897 | (0.82, 0.98) | 0.0186 | * |
|  | Single (never married) | 1.022 | (0.94, 1.11) | 0.5826 |  |
|  | Unknown | 0.884 | (0.77, 1.01) | 0.0756 |  |
|  | Widowed | 0.911 | (0.82, 1.02) | 0.1041 |  |
|  | Married (including common law) | Reference |  |  |  |
| **Race/ethnicity** | | | | | |
|  | Hispanic | 1.024 | (0.92, 1.14) | 0.6672 |  |
|  | Non-Hispanic Black | 1.199 | (1.08, 1.33) | 0.0007 | *** |
|  | Non-Hispanic Other | 1.066 | (0.89, 1.29) | 0.4980 |  |
|  | Non-Hispanic White | Reference |  |  |  |
| **Year of diagnosis** | | | | | |
|  | 2008-2011 | 0.873 | (0.79, 0.96) | 0.0069 | ** |
|  | 2012-2015 | 0.859 | (0.78, 0.95) | 0.0020 | ** |
|  | 2016-2020 | 0.743 | (0.68, 0.81) | <.0001 | *** |
|  | 2004-2007 | Reference |  |  |  |
| **Rural-urban continuum code** | | | | | |
|  | Metro 250,000 to 1 million | 1.022 | (0.95, 1.11) | 0.5774 |  |
|  | Metro <250,000 | 1.016 | (0.90, 1.14) | 0.7878 |  |
|  | Nonmetropolitan adjacent | 1.063 | (0.94, 1.20) | 0.3185 |  |
|  | Nonmetropolitan nonadjacent | 0.878 | (0.76, 1.02) | 0.0814 |  |
|  | Metro >=1 million | Reference |  |  |  |
| **T classification** | | | | | |
|  | T1 | 2.826 | (2.61, 3.07) | <.0001 | *** |
|  | T3 | 0.676 | (0.61, 0.75) | <.0001 | *** |
|  | T4 | 0.634 | (0.55, 0.73) | <.0001 | *** |
|  | TX | 1.095 | (1.01, 1.19) | 0.0284 | * |
|  | T2 | Reference |  |  |  |
| **N classification** | | | | | |
|  | N1 | 0.460 | (0.42, 0.51) | <.0001 | *** |
|  | N2 | 0.471 | (0.42, 0.53) | <.0001 | *** |
|  | N3 | 0.322 | (0.27, 0.38) | <.0001 | *** |
|  | NX | 0.791 | (0.68, 0.92) | 0.0032 | ** |
|  | N0 | Reference |  |  |  |
| **Subsite category** | | | | | |
|  | Anus, NOS or cloacogenic zone | 1.386 | (1.30, 1.48) | <.0001 | *** |
|  | Overlapping lesion of rectum, anus, and anal canal | 1.196 | (1.08, 1.32) | 0.0004 | *** |
|  | Anal canal | Reference |  |  |  |

** P<0.05; ** P<0.01; *** P<0.001.*

**Supplementary Table S4. Multivariable Cox proportional hazards model for overall survival in the sensitivity analysis including T1N0 cases.**

| **Factor** | **Level** | **Hazard ratio** | **95% CI** | **P value** | **Sig** |
| --- | --- | --- | --- | --- | --- |
| **Age** | | | | | |
|  | 50-59 years | 0.78 | [0.73, 0.84] | <0.001 | *** |
|  | 70-79 years | 1.69 | [1.57, 1.81] | <0.001 | *** |
|  | 80+ years | 3.34 | [3.10, 3.61] | <0.001 | *** |
|  | <50 years | 0.66 | [0.61, 0.72] | <0.001 | *** |
|  | 60-69 years | Reference |  |  |  |
| **Sex** | | | | | |
|  | Male | 1.56 | [1.48, 1.64] | <0.001 | *** |
|  | Female | Reference |  |  |  |
| **Marital status** | | | | | |
|  | Divorced, separated, unmarried | 1.37 | [1.28, 1.47] | <0.001 | *** |
|  | Single (never married) | 1.39 | [1.30, 1.48] | <0.001 | *** |
|  | Unknown | 1.07 | [0.96, 1.20] | 0.241 |  |
|  | Widowed | 1.52 | [1.41, 1.64] | <0.001 | *** |
|  | Married (including common law) | Reference |  |  |  |
| **Race/ethnicity** | | | | | |
|  | Hispanic | 0.98 | [0.90, 1.07] | 0.728 |  |
|  | Non-Hispanic Black | 1.28 | [1.19, 1.38] | <0.001 | *** |
|  | Non-Hispanic Other | 0.74 | [0.63, 0.87] | <0.001 | *** |
|  | Non-Hispanic White | Reference |  |  |  |
| **Year of diagnosis** | | | | | |
|  | 2008-2011 | 0.95 | [0.89, 1.01] | 0.092 |  |
|  | 2012-2015 | 0.88 | [0.82, 0.94] | <0.001 | *** |
|  | 2016-2020 | 0.78 | [0.72, 0.84] | <0.001 | *** |
|  | 2004-2007 | Reference |  |  |  |
| **Rural-urban continuum code** | | | | | |
|  | Metro 250,000 to 1 million | 1.12 | [1.05, 1.18] | <0.001 | *** |
|  | Metro <250,000 | 1.20 | [1.10, 1.30] | <0.001 | *** |
|  | Nonmetropolitan adjacent | 1.17 | [1.06, 1.28] | 0.001 | ** |
|  | Nonmetropolitan nonadjacent | 1.21 | [1.08, 1.35] | 0.001 | ** |
|  | Metro >=1 million | Reference |  |  |  |
| **T classification** | | | | | |
|  | T1 | 0.68 | [0.63, 0.73] | <0.001 | *** |
|  | T3 | 1.45 | [1.35, 1.56] | <0.001 | *** |
|  | T4 | 1.75 | [1.60, 1.90] | <0.001 | *** |
|  | TX | 1.17 | [1.10, 1.24] | <0.001 | *** |
|  | T2 | Reference |  |  |  |
| **N classification** | | | | | |
|  | N1 | 1.15 | [1.06, 1.25] | 0.001 | ** |
|  | N2 | 1.25 | [1.15, 1.35] | <0.001 | *** |
|  | N3 | 1.52 | [1.38, 1.67] | <0.001 | *** |
|  | NX | 1.45 | [1.29, 1.63] | <0.001 | *** |
|  | N0 | Reference |  |  |  |
| **Subsite category** | | | | | |
|  | Anus, NOS or cloacogenic zone | 1.10 | [1.05, 1.16] | <0.001 | *** |
|  | Overlapping lesion of rectum, anus, and anal canal | 1.16 | [1.08, 1.25] | <0.001 | *** |
|  | Anal canal | Reference |  |  |  |

** P<0.05; ** P<0.01; *** P<0.001.*

**Supplementary Analysis B. Local excision versus no surgery (excluding APR cases)**

This section reports the sensitivity analysis in which procedures classified as abdominoperineal resection were excluded and local excision was compared with no surgery.

**Supplementary Table S5. Cohort characteristics for the local excision versus no-surgery sensitivity analysis (excluding APR cases; N=15,853).**

| **Characteristic** | **Value** |
| --- | --- |
| **Age, n (%)** | |
| 50-59 years | 4541 (28.6%) |
| 60-69 years | 4525 (28.5%) |
| 70-79 years | 2615 (16.5%) |
| 80+ years | 1629 (10.3%) |
| <50 years | 2543 (16.0%) |
| **Sex, n (%)** | |
| Female | 10278 (64.8%) |
| Male | 5575 (35.2%) |
| **Marital status, n (%)** | |
| Divorced, separated, unmarried | 2628 (16.6%) |
| Married (including common law) | 6139 (38.7%) |
| Single (never married) | 4306 (27.2%) |
| Unknown | 955 (6.0%) |
| Widowed | 1825 (11.5%) |
| **Race/ethnicity, n (%)** | |
| Hispanic | 1388 (8.8%) |
| Non-Hispanic White | 12461 (78.6%) |
| Non-Hispanic Black | 1565 (9.9%) |
| Non-Hispanic Other | 439 (2.8%) |
| **Year of diagnosis, n (%)** | |
| 2004 | 546 (3.4%) |
| 2005 | 642 (4.0%) |
| 2006 | 617 (3.9%) |
| 2007 | 704 (4.4%) |
| 2008 | 770 (4.9%) |
| 2009 | 834 (5.3%) |
| 2010 | 858 (5.4%) |
| 2011 | 835 (5.3%) |
| 2012 | 903 (5.7%) |
| 2013 | 972 (6.1%) |
| 2014 | 1043 (6.6%) |
| 2015 | 1092 (6.9%) |
| 2016 | 1117 (7.0%) |
| 2017 | 1115 (7.0%) |
| 2018 | 1263 (8.0%) |
| 2019 | 1290 (8.1%) |
| 2020 | 1252 (7.9%) |
| **Local excision, n (%)** | |
| Yes | 4664 (29.4%) |
| No | 11189 (70.6%) |
| **Radiation, n (%)** | |
| No | 2369 (14.9%) |
| Yes | 13484 (85.1%) |
| **Rural-urban continuum code, n (%)** | |
| Metro >=1 million | 9487 (59.8%) |
| Metro 250,000 to 1 million | 3265 (20.6%) |
| Metro <250,000 | 1235 (7.8%) |
| Nonmetropolitan adjacent | 1101 (6.9%) |
| Nonmetropolitan nonadjacent | 765 (4.8%) |
| **T classification, n (%)** | |
| T1 | 591 (3.7%) |
| T2 | 6975 (44.0%) |
| T3 | 2500 (15.8%) |
| T4 | 1392 (8.8%) |
| TX | 4395 (27.7%) |
| **N classification, n (%)** | |
| N0 | 9694 (61.1%) |
| N1 | 2713 (17.1%) |
| N2 | 1652 (10.4%) |
| N3 | 1015 (6.4%) |
| NX | 779 (4.9%) |
| **Subsite category, n (%)** | |
| Anus, NOS or cloacogenic zone | 6602 (41.6%) |
| Anal canal | 7388 (46.6%) |
| Overlapping lesion of rectum, anus, and anal canal | 1863 (11.8%) |

**Supplementary Table S6. Patient characteristics by receipt of local excision versus no surgery in the sensitivity analysis excluding APR cases.**

| **Characteristic** | **Level** | **Yes** | **No** | **P value** |
| --- | --- | --- | --- | --- |
| **Age, n (%)** | | | | |
|  | 50-59 years | 1321 (29.1%) | 3220 (70.9%) | <.0001 |
|  | 60-69 years | 1224 (27.0%) | 3301 (73.0%) |  |
|  | 70-79 years | 704 (26.9%) | 1911 (73.1%) |  |
|  | 80+ years | 404 (24.8%) | 1225 (75.2%) |  |
|  | <50 years | 1011 (39.8%) | 1532 (60.2%) |  |
| **Sex, n (%)** | | | | |
|  | Female | 2637 (25.7%) | 7641 (74.3%) | <.0001 |
|  | Male | 2027 (36.4%) | 3548 (63.6%) |  |
| **Marital status, n (%)** | | | | |
|  | Divorced, separated, unmarried | 673 (25.6%) | 1955 (74.4%) | <.0001 |
|  | Married (including common law) | 1822 (29.7%) | 4317 (70.3%) |  |
|  | Single (never married) | 1449 (33.7%) | 2857 (66.3%) |  |
|  | Unknown | 283 (29.6%) | 672 (70.4%) |  |
|  | Widowed | 437 (23.9%) | 1388 (76.1%) |  |
| **Race/ethnicity, n (%)** | | | | |
|  | Hispanic | 414 (29.8%) | 974 (70.2%) | 0.0001 |
|  | Non-Hispanic White | 3582 (28.7%) | 8879 (71.3%) |  |
|  | Non-Hispanic Black | 536 (34.2%) | 1029 (65.8%) |  |
|  | Non-Hispanic Other | 132 (30.1%) | 307 (69.9%) |  |
| **Year of diagnosis, n (%)** | | | | |
|  | 2004 | 232 (42.5%) | 314 (57.5%) | <.0001 |
|  | 2005 | 239 (37.2%) | 403 (62.8%) |  |
|  | 2006 | 207 (33.5%) | 410 (66.5%) |  |
|  | 2007 | 243 (34.5%) | 461 (65.5%) |  |
|  | 2008 | 262 (34.0%) | 508 (66.0%) |  |
|  | 2009 | 263 (31.5%) | 571 (68.5%) |  |
|  | 2010 | 255 (29.7%) | 603 (70.3%) |  |
|  | 2011 | 245 (29.3%) | 590 (70.7%) |  |
|  | 2012 | 271 (30.0%) | 632 (70.0%) |  |
|  | 2013 | 295 (30.3%) | 677 (69.7%) |  |
|  | 2014 | 335 (32.1%) | 708 (67.9%) |  |
|  | 2015 | 280 (25.6%) | 812 (74.4%) |  |
|  | 2016 | 271 (24.3%) | 846 (75.7%) |  |
|  | 2017 | 276 (24.8%) | 839 (75.2%) |  |
|  | 2018 | 330 (26.1%) | 933 (73.9%) |  |
|  | 2019 | 339 (26.3%) | 951 (73.7%) |  |
|  | 2020 | 321 (25.6%) | 931 (74.4%) |  |
| **Radiation, n (%)** | | | | |
|  | No | 1345 (56.8%) | 1024 (43.2%) | <.0001 |
|  | Yes | 3319 (24.6%) | 10165 (75.4%) |  |
| **Rural-urban continuum code, n (%)** | | | | |
|  | Metro >=1 million | 2830 (29.8%) | 6657 (70.2%) | 0.4812 |
|  | Metro 250,000 to 1 million | 950 (29.1%) | 2315 (70.9%) |  |
|  | Metro <250,000 | 358 (29.0%) | 877 (71.0%) |  |
|  | Nonmetropolitan adjacent | 322 (29.2%) | 779 (70.8%) |  |
|  | Nonmetropolitan nonadjacent | 204 (26.7%) | 561 (73.3%) |  |
| **T classification, n (%)** | | | | |
|  | T1 | 273 (46.2%) | 318 (53.8%) | <.0001 |
|  | T2 | 2141 (30.7%) | 4834 (69.3%) |  |
|  | T3 | 443 (17.7%) | 2057 (82.3%) |  |
|  | T4 | 153 (11.0%) | 1239 (89.0%) |  |
|  | TX | 1654 (37.6%) | 2741 (62.4%) |  |
| **N classification, n (%)** | | | | |
|  | N0 | 3418 (35.3%) | 6276 (64.7%) | <.0001 |
|  | N1 | 453 (16.7%) | 2260 (83.3%) |  |
|  | N2 | 373 (22.6%) | 1279 (77.4%) |  |
|  | N3 | 143 (14.1%) | 872 (85.9%) |  |
|  | NX | 277 (35.6%) | 502 (64.4%) |  |
| **Subsite category, n (%)** | | | | |
|  | Anus, NOS or cloacogenic zone | 2228 (33.7%) | 4374 (66.3%) | <.0001 |
|  | Anal canal | 1936 (26.2%) | 5452 (73.8%) |  |
|  | Overlapping lesion of rectum, anus, and anal canal | 500 (26.8%) | 1363 (73.2%) |  |

*Abbreviation: APR, abdominoperineal resection. P values from chi-square tests.*

**Supplementary Table S7. Multivariable logistic regression for receipt of local excision versus no surgery in the sensitivity analysis excluding APR cases.**

| **Factor** | **Level** | **Odds ratio** | **95% CI** | **P value** | **Sig** |
| --- | --- | --- | --- | --- | --- |
| **Age** | | | | | |
|  | 50-59 years | 1.060 | (0.96, 1.17) | 0.2334 |  |
|  | 70-79 years | 0.993 | (0.89, 1.11) | 0.9102 |  |
|  | 80+ years | 0.845 | (0.73, 0.97) | 0.0204 | * |
|  | <50 years | 1.607 | (1.44, 1.80) | <.0001 | *** |
|  | 60-69 years | Reference |  |  |  |
| **Sex** | | | | | |
|  | Male | 1.438 | (1.33, 1.56) | <.0001 | *** |
|  | Female | Reference |  |  |  |
| **Marital status** | | | | | |
|  | Divorced, separated, unmarried | 0.853 | (0.77, 0.95) | 0.0042 | ** |
|  | Single (never married) | 0.977 | (0.89, 1.08) | 0.6436 |  |
|  | Unknown | 0.847 | (0.72, 0.99) | 0.0408 | * |
|  | Widowed | 0.856 | (0.75, 0.98) | 0.0244 | * |
|  | Married (including common law) | Reference |  |  |  |
| **Race/ethnicity** | | | | | |
|  | Hispanic | 1.002 | (0.88, 1.14) | 0.9704 |  |
|  | Non-Hispanic Black | 1.170 | (1.04, 1.32) | 0.0120 | * |
|  | Non-Hispanic Other | 1.085 | (0.87, 1.35) | 0.4642 |  |
|  | Non-Hispanic White | Reference |  |  |  |
| **Year of diagnosis** | | | | | |
|  | 2008-2011 | 0.839 | (0.75, 0.94) | 0.0029 | ** |
|  | 2012-2015 | 0.873 | (0.78, 0.98) | 0.0180 | * |
|  | 2016-2020 | 0.758 | (0.68, 0.85) | <.0001 | *** |
|  | 2004-2007 | Reference |  |  |  |
| **Rural-urban continuum code** | | | | | |
|  | Metro 250,000 to 1 million | 1.036 | (0.95, 1.14) | 0.4422 |  |
|  | Metro <250,000 | 0.991 | (0.86, 1.14) | 0.9037 |  |
|  | Nonmetropolitan adjacent | 1.037 | (0.90, 1.20) | 0.6152 |  |
|  | Nonmetropolitan nonadjacent | 0.850 | (0.71, 1.01) | 0.0687 |  |
|  | Metro >=1 million | Reference |  |  |  |
| **T classification** | | | | | |
|  | T1 | 3.491 | (2.90, 4.21) | <.0001 | *** |
|  | T3 | 0.547 | (0.49, 0.62) | <.0001 | *** |
|  | T4 | 0.358 | (0.30, 0.43) | <.0001 | *** |
|  | TX | 1.181 | (1.09, 1.29) | 0.0001 | *** |
|  | T2 | Reference |  |  |  |
| **N classification** | | | | | |
|  | N1 | 0.374 | (0.33, 0.42) | <.0001 | *** |
|  | N2 | 0.474 | (0.42, 0.54) | <.0001 | *** |
|  | N3 | 0.313 | (0.26, 0.38) | <.0001 | *** |
|  | NX | 0.786 | (0.67, 0.93) | 0.0044 | ** |
|  | N0 | Reference |  |  |  |
| **Subsite category** | | | | | |
|  | Anus, NOS or cloacogenic zone | 1.366 | (1.27, 1.48) | <.0001 | *** |
|  | Overlapping lesion of rectum, anus, and anal canal | 1.126 | (1.00, 1.27) | 0.0533 |  |
|  | Anal canal | Reference |  |  |  |

** P<0.05; ** P<0.01; *** P<0.001.*

**Supplementary Table S8. Multivariable Cox proportional hazards model for overall survival in the sensitivity analysis excluding APR cases.**

| **Factor** | **Level** | **Hazard ratio** | **95% CI** | **P value** | **Sig** |
| --- | --- | --- | --- | --- | --- |
| **Age** | | | | | |
|  | 50-59 years | 0.79 | [0.73, 0.85] | <0.001 | *** |
|  | 70-79 years | 1.70 | [1.57, 1.84] | <0.001 | *** |
|  | 80+ years | 3.40 | [3.13, 3.70] | <0.001 | *** |
|  | <50 years | 0.66 | [0.61, 0.73] | <0.001 | *** |
|  | 60-69 years | Reference |  |  |  |
| **Sex** | | | | | |
|  | Male | 1.56 | [1.47, 1.65] | <0.001 | *** |
|  | Female | Reference |  |  |  |
| **Marital status** | | | | | |
|  | Divorced, separated, unmarried | 1.34 | [1.24, 1.45] | <0.001 | *** |
|  | Single (never married) | 1.38 | [1.29, 1.48] | <0.001 | *** |
|  | Unknown | 1.06 | [0.94, 1.20] | 0.354 |  |
|  | Widowed | 1.48 | [1.36, 1.61] | <0.001 | *** |
|  | Married (including common law) | Reference |  |  |  |
| **Race/ethnicity** | | | | | |
|  | Hispanic | 0.99 | [0.90, 1.09] | 0.786 |  |
|  | Non-Hispanic Black | 1.26 | [1.16, 1.37] | <0.001 | *** |
|  | Non-Hispanic Other | 0.71 | [0.58, 0.85] | <0.001 | *** |
|  | Non-Hispanic White | Reference |  |  |  |
| **Year of diagnosis** | | | | | |
|  | 2008-2011 | 0.94 | [0.88, 1.01] | 0.100 |  |
|  | 2012-2015 | 0.88 | [0.81, 0.95] | 0.001 | ** |
|  | 2016-2020 | 0.79 | [0.72, 0.86] | <0.001 | *** |
|  | 2004-2007 | Reference |  |  |  |
| **Rural-urban continuum code** | | | | | |
|  | Metro 250,000 to 1 million | 1.11 | [1.04, 1.19] | 0.001 | ** |
|  | Metro <250,000 | 1.22 | [1.11, 1.34] | <0.001 | *** |
|  | Nonmetropolitan adjacent | 1.17 | [1.06, 1.29] | 0.003 | ** |
|  | Nonmetropolitan nonadjacent | 1.21 | [1.08, 1.37] | 0.002 | ** |
|  | Metro >=1 million | Reference |  |  |  |
| **T classification** | | | | | |
|  | T1 | 0.73 | [0.61, 0.87] | <0.001 | *** |
|  | T3 | 1.43 | [1.33, 1.54] | <0.001 | *** |
|  | T4 | 1.73 | [1.58, 1.90] | <0.001 | *** |
|  | TX | 1.18 | [1.11, 1.26] | <0.001 | *** |
|  | T2 | Reference |  |  |  |
| **N classification** | | | | | |
|  | N1 | 1.09 | [1.00, 1.19] | 0.063 |  |
|  | N2 | 1.25 | [1.15, 1.36] | <0.001 | *** |
|  | N3 | 1.54 | [1.40, 1.69] | <0.001 | *** |
|  | NX | 1.45 | [1.29, 1.64] | <0.001 | *** |
|  | N0 | Reference |  |  |  |
| **Subsite category** | | | | | |
|  | Anus, NOS or cloacogenic zone | 1.10 | [1.04, 1.16] | 0.001 | ** |
|  | Overlapping lesion of rectum, anus, and anal canal | 1.17 | [1.07, 1.27] | <0.001 | *** |
|  | Anal canal | Reference |  |  |  |

** P<0.05; ** P<0.01; *** P<0.001.*
